# Left–right asymmetry: cilia stir up new surprises in the node

**DOI:** 10.1098/rsob.130052

**Published:** 2013-05

**Authors:** Deepak Babu, Sudipto Roy

**Affiliations:** 1 Institute of Molecular and Cell Biology, Proteos, 61 Biopolis Drive, Singapore 138673, Republic of Singapore; 2 NUS Graduate School of Integrative Sciences and Engineering, Centre for Life Sciences, National University of Singapore, 28 Medical Drive, Singapore 117456, Republic of Singapore; 3 Department of Biological Sciences, National University of Singapore, 14 Science Drive 4, Singapore 117543, Republic of Singapore; 4 School of Biological Sciences, Nanyang Technological University, 60 Nanyang Drive, Singapore 637551, Republic of Singapore

**Keywords:** left–right asymmetry, node, motile cilia, immotile cilia, Pkd2, calcium signalling

## Abstract

Cilia are microtubule-based hair-like organelles that project from the surface of most eukaryotic cells. They play critical roles in cellular motility, fluid transport and a variety of signal transduction pathways. While we have a good appreciation of the mechanisms of ciliary biogenesis and the details of their structure, many of their functions demand a more lucid understanding. One such function, which remains as intriguing as the time when it was first discovered, is how beating cilia in the node drive the establishment of left–right asymmetry in the vertebrate embryo. The bone of contention has been the two schools of thought that have been put forth to explain this phenomenon. While the ‘morphogen hypothesis’ believes that ciliary motility is responsible for the transport of a morphogen preferentially to the left side, the ‘two-cilia model’ posits that the motile cilia generate a leftward-directed fluid flow that is somehow sensed by the immotile sensory cilia on the periphery of the node. Recent studies with the mouse embryo argue in favour of the latter scenario. Yet this principle may not be generally conserved in other vertebrates that use nodal flow to specify their left–right axis. Work with the teleost fish medaka raises the tantalizing possibility that motility as well as sensory functions of the nodal cilia could be residing within the same organelle. In the end, how ciliary signalling is transmitted to institute asymmetric gene expression that ultimately induces asymmetric organogenesis remains unresolved.

## 2. Introduction

Cilia can be classified as immotile or motile based on ultrastructural and functional differences [1]. Immotile or primary cilia are typically short and are differentiated by most cell types in the vertebrates. The principal role of the immotile cilia is in providing a platform for the transduction of a variety of morphogenetic and sensory signals that are decisive for embryonic development as well as adult physiology. Motile cilia, on the other hand, are longer, and the presence of dynein arms confers them with the ability to beat. Consequently, motile cilia are crucial for cellular (sperm) and organismal motility (many species of protozoans and larval forms of many invertebrates) as well as in the generation of fluid-flow over epithelia, such as mucus clearance in the respiratory tract and circulation of cerebrospinal fluid within the brain and spinal cord. However, a number of studies have uncovered that the motile cilia also possess sensory modalities, which means that the functional differences between the two kinds of cilia are, in fact, not as strictly demarcated [2].

**Figure 1.**
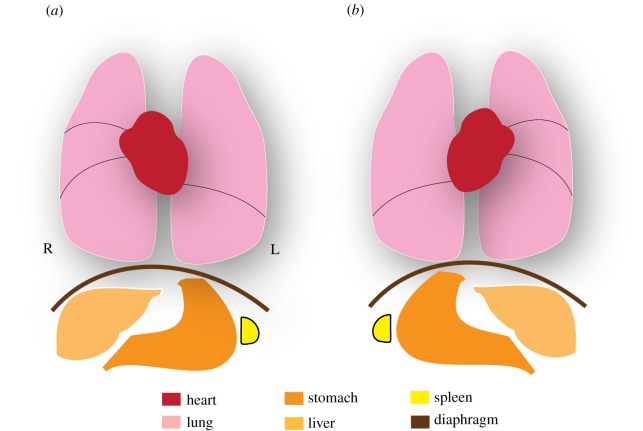
Left–right (L–R) asymmetry in man. (*a*) In the wild-type, also known as ‘situs solitus’, the heart, stomach and spleen are oriented to the left side, whereas the liver is present on the right side. (*b*) In KS patients with ‘situs inversus’, transposition of the visceral organs occurs in a mirror-image along the L–R axis. R indicates the right side, while L indicates the left side.

**Figure 2.**
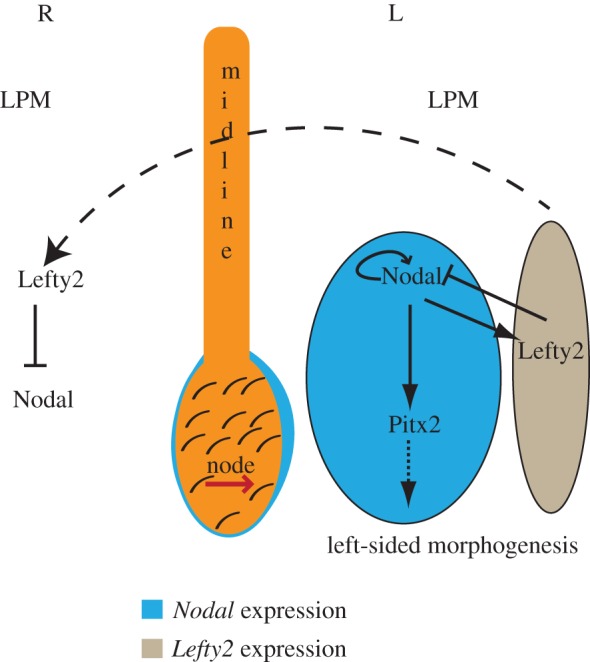
Nodal pathway activity in the determination of L–R asymmetry. A simplified schematic depicting asymmetric *Nodal* expression in the node, and the essential elements of asymmetric Nodal signalling in the left LPM.

A conserved theme in vertebrate development is the generation of sidedness (left versus right) with reference to the embryonic midline, which eventually gets translated into the asymmetric disposition of visceral organs or situs apparent in the adult. Thus, even though exteriorly the human body is bilaterally symmetric, internally the apex of the heart, the stomach and the spleen invariably lie to the left, whereas the liver is always situated on the right (figure 1*a*). How an otherwise bilaterally symmetric embryo can distinguish left from right and then organize the positioning of organs in the appropriate directions is a conceptually challenging problem. Left–right (L–R) asymmetries in embryos actually arise much earlier than the morphological asymmetries of the visceral organs. In the mid-1990s, work from several groups revealed that asymmetric expression of genes during early embryogenesis presaged the development of morphological asymmetry of the vertebrate body [3–6]. In the mouse embryo, one such gene, *Nodal*, is initially expressed throughout the node, a transient embryonic cavity that forms at the end of the developing notochord, and then becomes restricted to the left side of the node [5–7] (figure 2). The left-sided expression of *Nodal*, which encodes a member of the transforming growth factor beta (TGFβ) family of secreted signalling proteins, then spreads out to the tissue adjacent to the node, the lateral plate mesoderm (LPM), where Nodal induces its own expression, as well as that of *Lefty2* and *Pitx2*. Like *Nodal*, *Lefty2* encodes another member of the TGFβ family that competitively binds to a class of Nodal receptors. Biochemically, Lefty2 exists as a monomer, unlike Nodal, which functions as a dimer. This property enables Lefty2 to diffuse faster and farther than Nodal, thereby limiting the influence of Nodal activity to the left side. On the other hand, Pitx2, a paired-like homeodomain transcription factor, is the effector of Nodal signalling. Pitx2 is thought to dictate the subsequent asymmetric morphogenetic events by regulating the gene expression programme important for left-sided morphogenesis [8].

But how does the asymmetric pattern of Nodal pathway genes get established in the first place? The issue became even more enigmatic with the possibility that leftward flow of extraembryonic fluid, driven by the rotary movement of monocilia that differentiate on the nodal cells, is what triggers the onset of L–R asymmetric gene expression [9] (figure 2). This provocative concept, termed nodal flow, immediately caught the imagination of developmental biologists, who began unravelling its mystery using genetics, sophisticated microscopy and biophysical approaches. Here, we briefly recount the important discoveries that have shaped the field, and then critically examine the current state of our understanding of the heart of the problem—the motility and sensory functions of cilia in the generation and perception of nodal flow.

## 3. Cilia and left–right asymmetry: the origins

The connection between cilia and L–R asymmetry has its origins in the mid-1970s and, fascinatingly, through studies of human patients afflicted with a very rare genetic disorder called Kartagener syndrome (KS) [10,11]. Classical presentation of this disease includes respiratory dysfunction such as chronic rhinosinusitis and bronchiectasis together with ‘situs inversus’, wherein there is a mirror-image reversal in the orientation of visceral organs (figure 1*b*). Although the respiratory insufficiency of these patients could be correlated with ultrastructural defects in their airway motile cilia [10,11], how ciliary abnormalities could be responsible for the incorrect positioning of visceral organs remained a confounding problem for a very long time. In fact, the solution came about 20 years later, rather serendipitously, from the analysis of genetically engineered mice that were deficient in genes encoding kinesin proteins. Kinesins, which are enzymes that track along microtubules in an ATP-dependent manner and participate in the trafficking of a variety of cargoes within the cell, are also required for the assembly of cilia in a process called intraflagellar transport. Mice mutant for the kinesin genes *Kif3a* or *Kif3b* failed to assemble cilia, and, strikingly, approximately 50 per cent of the mutant embryos showed a reversal in L–R patterning, resembling patients afflicted with KS [9,12]. Indeed, the expression of *Lefty2* in the LPM was disrupted, signifying that the earliest molecular events in the determination of L–R asymmetry were affected. Direct visualization of the node in wild-type embryos revealed motile monocilia that beat in a clockwise rotary pattern (when viewed from the ventral side) to drive a leftward flow of extraembryonic fluid, whereas cilia and directional fluid flow were completely absent in the *Kif* mutant embryos [9,12,13]. These remarkable observations led to the formulation that cilia-driven nodal flow is an essential epigenetic cue that initiates L–R asymmetry. This view was strengthened by work from Supp *et al*. [14] through the analysis of the *inversus viscerum* (*iv*) mutant mice. The *iv* locus encodes a member of the dynein family—left–right dynein (Lrd), a protein that is required for ciliary motility [14]. Cilia were specified normally in the *iv* mutant mice, but the deficiency of Lrd rendered them immotile: the failure to institute a leftward flow then translated to a randomization of L–R asymmetry [13,15]. An even more persuasive finding that further bolstered the concept of nodal flow was the dramatic demonstration that asymmetries could be controlled *ex utero* by inducing flow exogenously. In a technologically challenging feat, Nonaka *et al*. [16] subjected cultured mouse embryos to artificial flow and made a stunning observation. The embryos not only responded to the flow, but asymmetries could even be reversed when a strong right-sided flow was introduced. Also, application of external flow restored situs in *iv* mutant embryos, which otherwise would have developed randomized asymmetry. But how do the rotating nodal cilia drive unidirectional fluid flow in the node?

## 4. Rotary beating of posteriorly tilted nodal monocilia produces leftward flow

The prototypical motile cilium, which beats in a planar whip-like pattern, contains a central pair (CP) of singlet microtubules in its axoneme, in addition to the nine doublet of peripheral microtubules (the 9 + 2 arrangement). Observation by Bellomo *et al*. [17] that nodal cilia lack the CP of microtubules (9 + 0 arrangement) led to the speculation that the absence of the CP apparatus confers the rotary pattern of beating to the node cilia. However, several lines of evidence suggest that this reasoning is unlikely to be correct. Teleost fishes such as the zebrafish and medaka also use ciliary motility to establish L–R asymmetry [18–20]. In these species, motile cilia reside in Kupffer's vesicle (KV), an organ of laterality that is the functional equivalent of the mammalian node. Although medaka KV cilia are 9 + 0 and beat in a rotary pattern [19], the zebrafish has CP-containing KV cilia (9 + 2 arrangement), and yet they exhibit rotational movement [20]. Likewise, contrary to the traditional view, it has been recently reported that the mouse node also contains cilia with the CP [21]. Thus, the presence or absence of the CP appears not to dictate the beating pattern of the cilium. However, genetic evidence does favour a view that the CP is dispensable for nodal cilia motility. In mice and humans, mutation of genes that are required for the assembly or function of the CP does not affect laterality, whereas the planar motility of other 9 + 2 cilia (such as those in the airways) is strongly affected [22,23]. Given these considerations, the mechanisms that confer rotary beat pattern to the nodal cilia remain an unresolved issue.

A more challenging problem is how rotary movement of the cilia could be linked to directional flow. At best, it would produce a vortex instead of biased flow. Here, theoretical fluid dynamics simulations of nodal cilia rotation could foretell that a linear directional flow will result if the rotational axes of the cilia are posteriorly tilted [24]. Such a tilt will ensure that the effective stroke (towards the left side) will be much more efficient than the recovery stroke (towards the right side), because the latter will have to move fluid much closer to the cell surface, where viscosity is higher. Experimental veracity of this prediction first came from a careful analysis of the dynamics of ciliary beat. Using high-speed videomicroscopy, two different groups found that the rotational axes of the nodal cilia are indeed tilted towards the posterior [19,25]. It was also observed that the surface of the nodal cells was conspicuously convex, and that the position of basal body, a derivative of the mother centriole to which cilia are anchored at the cell surface, shifted to the posterior side from an initial central location [19,25]. Even more impressively, Nonaka *et al*. [25] showed that motorized ‘artificial cilia’ could indeed drive a net leftward flow of viscous liquid silicone when their rotational axes were posteriorly tilted.

This leads us to question the mechanism by which the basal body transits to the posterior side of the nodal cells. Genetic studies with *Drosophila* had led to the discovery of the planar cell polarity (PCP) pathway that regulates the polarized morphology of a field of cells in the plane of an epithelium, such as the orientation of bristles on the cuticle of the fly [26]. PCP, like many conserved developmental pathways, also operates in vertebrates; for instance, during the polarized movement of cells during gastrulation. In fact, the connection between PCP and orientation of cilia was already appreciated in the context of the hair cells in the inner ear that are specialized to detect sound and linear acceleration. In the hair cell, the precise positioning of an immotile cilium, the kinocilium, is dependent on PCP [27]. Therefore, it was not too surprising when the PCP pathway was shown to be also required for the posterior migration of the basal bodies in the nodal cells [28–31]. Interestingly, many of the components of the PCP pathway—such as the protein Dishevelled (Dvl), Prickle (Pk) and Van gogh-like (Vangl)—have polarized subcellular localization in the node. While Vangl and Pk are found on the anterior side of the nodal cells, Dvl is found on the posterior [28–30,32]. Although it is now clear that the PCP pathway is behind the polarized orientation of the basal bodies, how the cues provided by PCP signalling are eventually translated into their directed posterior migration remains to be determined.

## 5. Making sense of nodal flow: the ‘morphogen’ hypothesis

**Figure 3.**
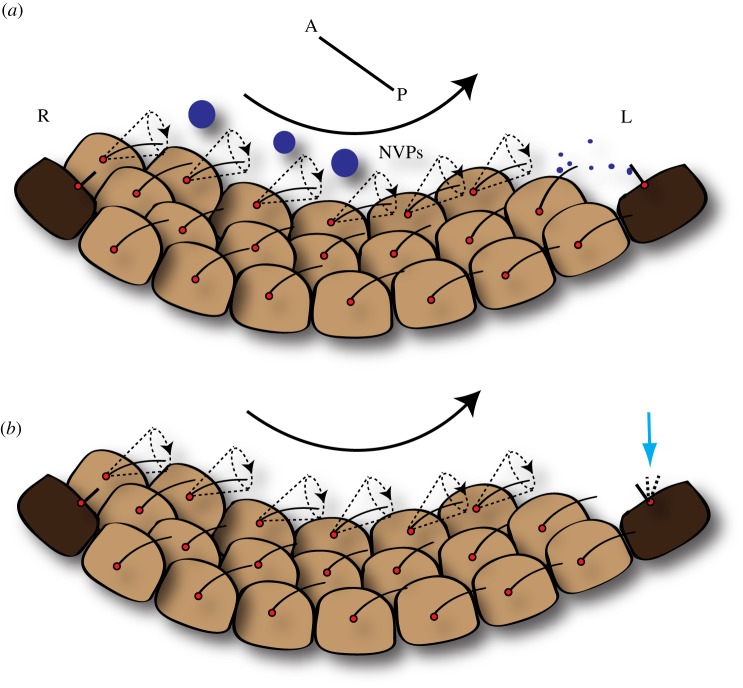
Models to explain the function of nodal flow in L–R asymmetry. (*a*) The ‘morphogen’ hypothesis. Clockwise beating of motile cilia transports a morphogen or NVPs towards the left side of the node. (*b*) The ‘two-cilia’ hypothesis. Fluid flow generated by the motile cilia is sensed by immotile cilia on the perinodal crown cells (shown here as deflections; blue arrow). Nodal pit cells are depicted in light brown, whereas perinodal crown cells are in dark brown. The motile cilia are tilted posteriorly. Basal bodies are indicated with red dots and the direction of nodal flow is shown with the black arrow. A, anterior; P, posterior.

Since the discovery of nodal flow, two hypotheses have evolved to explain how it functions in instituting L–R asymmetry. The first of these was termed the ‘morphogen hypothesis’. It proposes that directed beating of the nodal monocilia leads to a unidirectional transport of a secreted morphogen to the left side of the node [9,13]. This is a simplistic model, where ciliary beating ensures that one side of the node preferentially receives greater concentration of a morphogen than the other side (figure 3*a*). The asymmetry in the distribution of the morphogen then triggers signalling events that cement the asymmetry in the developing embryo. Although this hypothesis is appealing, it raises several questions, foremost among them being the identity of the morphogen itself. An important clue in this direction came from the work of Tanaka *et al*. [33]. The authors observed flowing material inside the node cavity. These particles, which they termed nodal vesicular parcels (NVPs), were seen to be released into the flow and to break upon contact with cilia, thereby emptying their contents on the left side of the node. They also provided some insight into the nature of the putative morphogen. They found that Sonic Hedgehog and Retinoic acid are ensheathed into the NVPs, and are released into the nodal flow in a fibroblast growth factor (FGF)-signalling-dependent manner [33]. Despite these captivating findings, the NVP model of the ‘morphogen hypothesis’ has not been further corroborated. Most importantly, genetic analysis of Sonic Hedgehog and the Retinoic acid pathways do not provide convincing support of their roles as nodal morphogens (see [34,35]).

## 6. Cilia generate as well as sense nodal flow: the ‘two-cilia hypothesis’

The clue that cilia could be involved not only in the generation but also the sensation of nodal flow came from the genetic analysis of mice mutant for Pkd2 [36], a Ca^2+^ permeable ion channel involved in the pathogenesis of autosomal dominant polycystic kidney disease (ADPKD) in man [36–38]. PKD2 physically interacts with another protein, PKD1, which is also mutated in ADPKD, and together they localize on immotile primary cilia of kidney tubule cells where they sense fluid-flow-induced mechanical stress [39–41]. PKD1 is an 11-pass transmembrane protein of 4302 amino acids, with a large N-terminal extracellular domain constituted by approximately 3000 amino acids [42,43]. By contrast, PKD2 is a much smaller 6-pass transmembrane protein, containing 968 amino acids [42]. *Pkd2* mutant mice displayed many features that typify aberrations in L–R asymmetry, thus implicating Ca^2+^ signalling in the establishment of L–R asymmetry [36]. Further support of this possibility came from the examination of Pkd2 localization on nodal cilia. While Lrd, the dynein protein required for ciliary motility, localized to the motile cilia on the central pit cells of the node, Pkd2 was present on the motile cilia as well as the immotile Lrd-negative cilia on the perinodal cells that surround the nodal pit [44]. This observation led to the birth of an alternative to the ‘morphogen hypothesis’, called the ‘two-cilia’ hypothesis. According to this view, beating of the motile cilia on pit cells at the centre of the node creates a leftward fluid flow, which is sensed by immotile cilia on the perinodal crown cells (figure 3*b*). Thus, the ‘two-cilia’ hypothesis adds an extra level of complexity to ciliary function in the node by partitioning the process of nodal flow into generating the flow and responding to the flow by two distinct kinds of cilia.

The involvement of Pkd2 activity in L–R asymmetry was further strengthened from the observation that Ca^2+^ signalling was preferentially elevated in the endodermal cells on the left side of the node [44]. Moreover, this asymmetric Ca^2+^ spike became randomized in the *iv* mutant mice, and was absent from those that lacked Pkd2 [44]. All of these findings favour the model where leftward fluid flow generated by the centrally located motile cilia in the pit cells is sensed by the peripherally located immotile cilia via Pkd2. This generates asymmetric Ca^2+^ signalling on the left, which then turns on the asymmetric expression of *Nodal* pathway genes.

Given that Pkd1 directly interacts with Pkd2, the Pkd1–Pkd2 complex localizes to primary cilia of renal cells, and the loss of *Pkd2* leads to defects in laterality, one would expect Pkd1 to also have a role in L–R asymmetry. Surprisingly, *Pkd1* is not expressed in the node, and in its absence, specification of L–R asymmetry is not perturbed [45]. How then does Pkd2 function in the node?

## 7. Advancing the story: discovery of Pkd1l1

The missing piece in the puzzle seems to be the protein encoded by *Pkd1l1*, a paralogue of Pkd1. Like Pdk1, Pkd1l1 is a large protein comprising 11 transmembrane segments, and C-terminal intracellular coiled-coil and N-terminal extracellular regions. *Pkd1l1* mutants displayed very striking L–R asymmetry defects such as inverted heart apex, inverted stomach situs and a fully penetrant right lung isomerism. Consistent with abnormalities in organ situs, expression of genes that presage the development of sidedness, such as *Nodal*, *Lefty2* and *Pitx2*, was affected [46]. Furthermore, *Pkd1l1* was found to be expressed quite specifically in the node, in a pattern that corresponded spatially and temporally to the establishment of L–R asymmetry. However, as in the *Pkd2* mutants, there were no alterations in the morphology of the node as well as in the number and motility of the nodal cilia in *Pkd1l1* mutant embryos. If Pkd1l1 is the partner of Pkd2 in the node, then, by analogy to the Pkd1–Pkd2 complex on kidney cilia, it is logical to expect that they would physically interact. Indeed, this is the case—Pkd1l1 and Pkd2 associate with each other through the C-terminal-coiled-coil domain of Pkd1l1, and this interaction appears to be necessary for the localization of the proteins to cilia [46]. This implies that, like Pkd2, Pkd1l1 also localizes to the motile as well as the non-motile cilia in the node. Thus, the discovery of *Pkd1l1*, together with earlier findings from the *Pkd2* mutant mice, strengthens the ‘two-cilia’ hypothesis by demonstrating that the ciliary beating to generate nodal flow can be genetically uncoupled from the ability of the embryo to sense nodal flow.

## 8. Generating and sensing nodal flow: a handful of cilia are sufficient

Where does the Pkd1l1–Pkd2 complex function—in the motile or the immotile cilia? And what do they sense in nodal flow—mechanical force or a morphogen? Before we examine the answers to these questions, it is important to consider one other study that has a significant bearing on our understanding of these issues. The mouse node is populated by approximately 200–300 motile cilia that produce a strong leftward laminar flow, and one would expect that most of these cilia, if not all, would be required to ensure the proper establishment of L–R asymmetry. However, the findings of Shinohara *et al*. [47] have challenged this notion. First, with the clever use of the viscous chemical methyl cellulose to retard ciliary movement in the node of cultured mouse embryos at specific developmental times, the authors discovered that a weak and transient local flow was adequate to initiate L–R asymmetric gene expression. Then, they studied embryos mutant for the ciliary genes *Rfx3* and *Dpcd*, in which the numbers of motile cilia were variably reduced, and found that as few as two beating cilia (greater than a 100-fold reduction from wild-type numbers!) are all that is required. Moreover, the geographical location of these cilia within the node cavity (i.e. in the centre of the cavity, to the left or to the right) had no consequence on the ability to correctly turn on asymmetric gene expression. Because these mutant embryos also had a significant reduction in the number of immotile cilia around the node, the overall conclusion is rather tantalizing: a very weak local leftward flow and just a small number immotile cilia are sufficient for the correct institution of L–R asymmetry.

In the context of the ‘morphogen’ versus the ‘two-cilia hypothesis’, these findings make a strong case for the validity of the latter. It is hard to conceive how a weak local flow would be sufficient to effectively transport a proteinaceous morphogen or the larger NVPs across the node. On the other hand, as Shinohara *et al*. [47] argue, because the mouse node is a semiclosed cavity (covered by Reichert's membrane), even a weak mechanical force produced by a few rotating cilia can, in theory, be transmitted instantaneously. Moreover, the immotile cilia are exquisitely sensitive to mechanical stress [41]. An attractive alternative is that the beating cilia themselves are able to sense the flow that they generate. If this is true, we will need to invoke the existence of a morphogen, because it is not easy to conceive how the directionality of flow could be mechanically sensed by rotating cilia. But before we explore such a scenario, we need to know which cells in the node actually perceive nodal flow.

## 9. Perinodal crown cells sense flow through Pkd2

**Figure 4.**
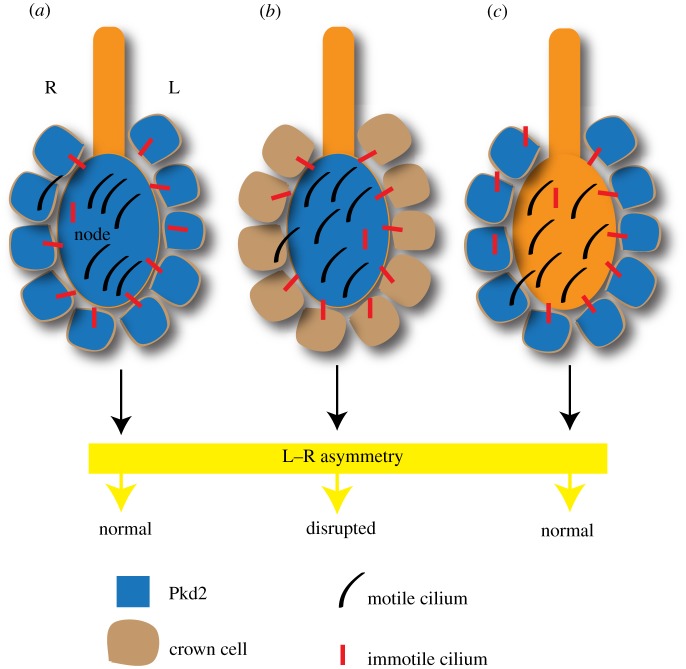
Crown cells sense nodal flow through Pkd2. (*a*) Pkd2 expression throughout the node (pit cells and crown cells) ensures correct inception of L–R asymmetry. The majority of pit cell cilia are motile, whereas the majority of crown cell cilia are immotile. (*b*) Pkd2 expression exclusively in the pit cells is not sufficient for the correct determination of L–R asymmetry. (*c*) Pkd2 expression only in crown cells is sufficient for proper determination of L–R asymmetry.

Using gene enhancers that are active in subsets of the nodal cells and elegant mouse genetics, Yoshiba *et al*. [48] were able to dissect the mouse node into distinct regions, and functionally analyse the consequences of the loss of Pkd2 in each of these regions. Interestingly, asymmetric Nodal pathway gene expression was rescued in *Pkd2* mutant embryos only when *Pkd2* was expressed either throughout the node or specifically in the crown cells, but not in the pit cells (figure 4*a–c*). As discussed in §8, the pit cells of the node cavity bear motile cilia, whereas the majority of the cilia on the crown cells are immotile. Furthermore, a mutant version of the Pkd2 protein that retained channel activity but was unable to localize to cilia failed to rescue L–R asymmetric gene expression in *Pkd2* mutant embryos. These data are significant because they define a spatial region within the node with a preponderance of immotile cilia where Pkd2 function is required to respond to nodal flow.

The results from the other experiments reported in this study, though illuminating, raise many new questions about nodal flow and L–R asymmetry. The first of these has to do with Ca^2+^ signalling. Because Pkd2 is a Ca^2+^ permeable ion channel, it was quite natural for the authors to investigate Ca^2+^ signalling in the crown cells. For this, they used a reporter transgene containing an enhancer of the human *LEFTY2* gene driving lacZ expression (*ANE-lacZ*). This transgene showed asymmetric expression in the crown cells of wild-type embryos, with higher levels on the left than the right (L > R). Because *Pkd2* mutants are unable to sense nodal flow, as expected, this asymmetric pattern of ANE activity was lost, and *lacZ* expression was observed bilaterally in the crown cells (L = R). Rather curiously, however, visualization of Ca^2+^ signalling in the crown cells of wild-type embryos (by transgenic expression of the Ca^2+^ indicator protein GCaMP2) did not reveal any asymmetry (L = R); even more perplexing was the observation that Ca^2+^ signalling levels in the crown cells of *Pkd2* mutants was completely unaffected. Despite these paradoxical findings, Ca^2+^ signalling inhibitors had a clear effect on *ANE* activity: asymmetric *lacZ* expression (L > R) became bilateral (L = R). Asymmetric Ca^2+^ signalling around the node that was reported in earlier studies occurs in the endoderm [33,44], which is distinct from the perinodal crown cells. Therefore, how Pkd2 activity and Ca^2+^ signalling set up asymmetric patterns of gene expression about the node remains an unresolved problem.

The second unexpected discovery that the authors made is that cilia on the crown cells are sufficient for the inception of L–R asymmetry. When *Kif3a* gene expression was introduced only in the crown cells (using the same crown cell enhancer that was used to express *Pkd2* in the crown cells of *Pkd2* mutants) of otherwise *Kif3a* mutant embryos, asymmetric *Nodal* expression in the left LPM was efficiently rescued. There was a weak leftward fluid flow in the node of these embryos, and some amount of local vortical flow. Even though cilia on the crown cells are predominantly immotile, there are some motile cilia as well. The authors interpret that the weak flow was produced by these motile cilia and was sensed by the neighbouring immotile cilia. Such a notion is consistent with the earlier finding that just a few motile cilia are sufficient for the correct establishment of L–R asymmetry [47]. Nevertheless, none of the experiments have ruled out the possibility that it is the motile cilia on the crown cells that not only generate, but also sense, nodal flow.

## 10. Do motile cilia generate and sense nodal flow?

**Figure 5.**
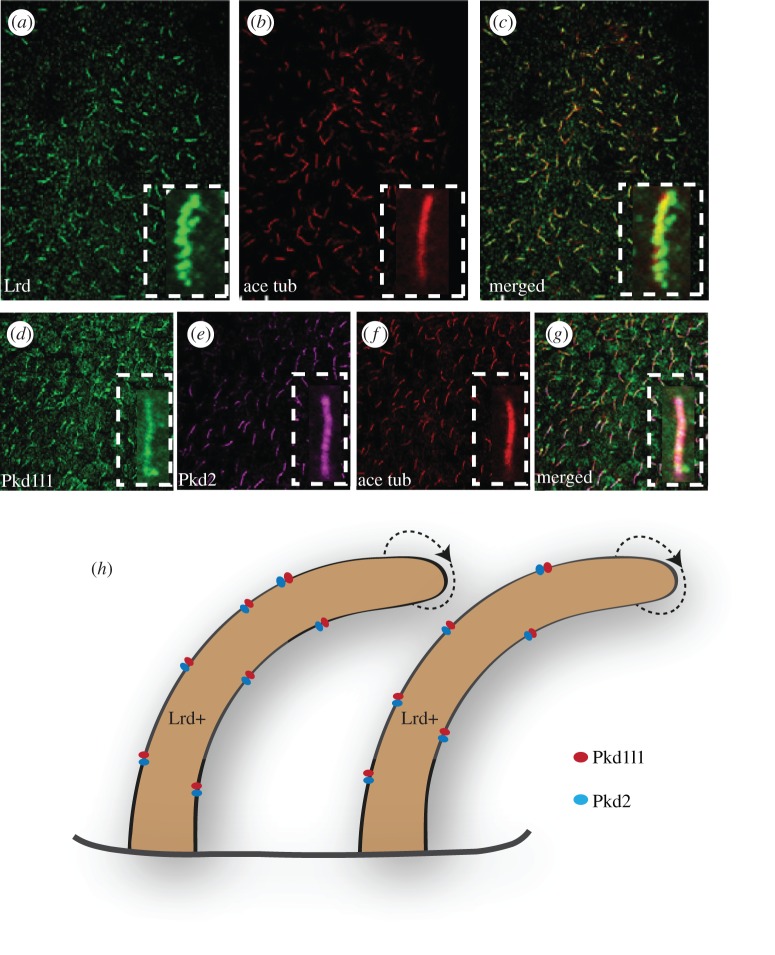
Motile cilia in the node may sense flow. (*a*) KV cilia of medaka fish embryo labelled with antibodies to Lrd. (*b*) KV cilia labelled with antibodies to acetylated tubulin (ace tub). (*c*) Merged image of (*a*,*b*) showing that all cilia in KV contain Lrd, and hence are motile. (*d*) KV cilia labelled with antibodies to Pkd1l1. (*e*) KV cilia labelled with antibodies to Pkd2. (*f*) KV cilia labelled with antibodies to acetylated tubulin. (*g*) Merged image of (*d–f*) showing that all cilia in KV contain Pkd1l1–Pkd2 complex. Insets in (*a–g*) highlight a single motile cilium. (*h*) showing that the cilia in medaka KV contain Lrd as well as the Pkd1l1–Pkd2 complex. (*a*–*g*) Reproduced from Kamura *et al*. [50] with permission from the Company of Biologists.

With the exception of the chick, cilia-driven nodal flow has been shown to be required for L–R determination in all vertebrate model organisms examined to date [19,20,49]. Consistent with this conservation, Pkd1l1 was found to be a partner of Pkd2 even in the medaka fish [50]. As in the mouse embryo, expression of the medaka *pkd1l1* gene during embryogenesis was specific, and restricted to KV, which, as noted earlier, functions like the mammalian node [50]. In addition, medaka embryos homozygous for a mutation of *pkd1l1* showed defective chirality in the positioning of internal organs. Again, similar to data from mouse, the number of cilia in KV, their length and their motility were not affected. However, what is strikingly distinct is the distribution of the motile versus the immotile cilia in the mouse node versus the medaka KV. All of the cilia in KV express Lrd, Pkd1l1 and Pkd2; consistent with this, direct visualization of ciliary motility revealed that all KV cilia are motile [50] (figure 5*a*–*g*). How then is nodal flow sensed in the medaka? The authors believe the cause to be chemical sensation (figure 5*h*). They argue that although Pkd1l1 has a large extracellular domain-like Pkd1, the degree of homology in this region is limited, indicating that Pkd1l1 could be involved in sensing something different from mechanical forces that are sensed by Pkd1. Indeed, Pkd-like proteins participate in the sensation of a variety of chemical signals in many different organisms, making this possibility not too improbable [51–54]. Of course, this means that we must then take the hunt for the chemical signal even more seriously.

## 11. Cilia, nodal flow and left–right asymmetry: some answers, many questions

Ever since the discovery of nodal flow, ingenious experiments and theoretical analyses have tried to tease out the mechanisms by which cilia function in the determination of L–R asymmetry. Although these approaches have been very instructive, it is clear from all of the studies examined in this review that several fundamental aspects of the process continue to remain elusive. First, we are still not sure whether nodal flow generates a mechanical signal or transports a chemical morphogen. This is not going to be easy to decipher, but we can do experiments that may provide circumstantial evidence in support for one alternative over the other. For instance, can *Pkd1* rescue the L–R defects of *Pkd1l1* mutants when expressed in the crown cells? If it does, we can keep our hopes high that the signal is mechanical. Next, the finding that a few motile cilia located on the crown cells is sufficient for proper asymmetric gene expression is fascinating. What needs to be resolved is how an effective leftward flow can be generated by the rotary beating of cilia in this situation, given that the crown cells have a completely different geometry (squamous epithelium) in comparison with the pit cells (columnar epithelium with convex apical surface). It also appears that just a few immotile cilia are sufficient to interpret this flow. The basis for this highly sensitive flow detection mechanism needs an explanation. And where exactly is the flow sensed—on the right side of the node, the left side or all around the crown cells? While asymmetric gene expression can be instituted by just a few motile and sensory cilia, does this mean they are also sufficient for the complete realization of L–R asymmetry, including the proper development of organ situs? What about the possibility that the motile cilia themselves sense the flow? For sure, the motile cilia in the nodal pit cells are not required for sensing, but the same cannot be said for those on the crown cells. A strategy to selectively eliminate the immotile cilia could be informative. This can be achieved by reintroducing *Kif3a* function in the node of *Kif3a* null mice using the promoter of *FoxJ1. FoxJ1* encodes a master regulator of motile cilia biogenesis [55–58], and its promoter is specifically active in cells that differentiate motile cilia [59,60]. In case of the medaka, the data at hand make a good case for a sensory function of KV motile cilia. Although this could point to evolutionary differences in the way nodal flow is sensed in different groups of vertebrates, a thorough cell biological analysis is required to completely rule out the existence of immotile cilia within or in the vicinity of KV. Finally, the inconsistencies in the data linking Pkd2, Ca^2+^ signalling and asymmetric gene expression need to be smoothed out. Ca^2+^ signalling levels in the crown cells are symmetric and were not affected when Pkd2 function was lost [48]. Clearly, then, symmetric Ca^2+^ signalling levels cannot explain the asymmetric pattern of gene expression in the crown cells. Does this mean that Pkd2 activity in the crown cells is not mediated via Ca^2+^ signalling? A mutant variant of Pkd2 that lacks channel activity but retains the ability to localize to cilia could be used to examine this possibility. Another solution to this problem may lie in the very recent work of Takao *et al*. [61], where the spatio-temporal levels and patterns of Ca^2+^ signalling in the perinodal cells of mouse embryos were very carefully monitored. Using an alternative Ca^2+^ indicator, Fura-PE3, the authors found that Ca^2+^ signalling in the node is actually much more dynamic than previously recognized: in the wild-type, Ca^2+^ spikes were initially symmetric about the node, and then later they became more frequent on the left side. By contrast, in *iv* mutant embryos, where ciliary motility is disrupted and there is no nodal flow, the average Ca^2+^ signal distribution was symmetric. Even more satisfyingly, the total Ca^2+^ signal frequency in *Pkd2* mutants was significantly lower and more symmetric compared with wild-type embryos. Based on these findings, Takao *et al*. favour the idea that Pkd2 regulates the frequency of Ca^2+^ signals, and it is the frequency and not just the spatial distribution of the Ca^2+^ signals that is critical for initiating L–R asymmetry [61]. At this moment, one cannot rule out the possibility that the discrepancies between the observations of Yoshiba *et al*. and Takao *et al*. may have arisen from the different methods (genetically encoded versus exogenously applied Ca^2+^ indicators) that were used to track Ca^2+^ signalling. In any case, though the model that Takao *et al*. propose is attractive, it will need to be further tested.

In conclusion, how nodal flow determines L–R asymmetry continues to remain an enduring problem in developmental biology. We can expect that over the coming years, a combination of sophisticated experiments as well as intuitive theoretical modelling in the mouse and other vertebrate species will provide us with a much better appreciation of this remarkable morphogenetic phenomenon.

## 12. Acknowledgements

We thank S. Choksi for discussion and critical reading of the manuscript, and the Company of Biologists for their permission to reproduce published images. D.B. is supported by a fellowship from the NUS Graduate School of Integrative Sciences and Engineering. S.R. is supported by the Agency for Science, Technology and Research (A*STAR) of Singapore.
